# Glutathione Quantification in Live Cells with Real-Time Imaging and Flow Cytometry

**DOI:** 10.1016/j.xpro.2020.100170

**Published:** 2020-11-14

**Authors:** Xiqian Jiang, Jianwei Chen, Meng C. Wang, Jin Wang

**Affiliations:** 1 Department of Pharmacology and Chemical Biology, Baylor College of Medicine, Houston, TX 77030, USA; 2 Department of Molecular and Human Genetics, Baylor College of Medicine, Houston, TX 77030, USA; 3 Department of Molecular and Cellular Biology, Baylor College of Medicine, Houston, TX 77030, USA; 4 Huffington Center on Aging, Baylor College of Medicine, Houston, TX 77030, USA; 5 Howard Hughes Medical Institute, Baylor College of Medicine, Houston, TX 77030, USA; 6 Center for Drug Discovery, Baylor College of Medicine, Houston, TX 77030, USA

**Keywords:** Cell Biology, Flow Cytometry/Mass Cytometry, Microscopy, Molecular/Chemical Probes

## Abstract

Glutathione (GSH) is a highly dynamic, high abundance molecule regulating redox homeostasis in most mammalian cells. Traditional methods could not achieve quantification of glutathione in live cells with high spatial and temporal resolution. Here, we provide protocols on how to use reversible reaction-based ratiometric fluorescent probes, RealThiol (RT) and its derivatives, to quantify GSH globally or in specific organelles. The protocols are applicable to cultured or harvested cells through confocal imaging and flow cytometry.

For complete details on the use and execution of this protocol, please refer to Chen et al. (2017) and Jiang et al. (2015, 2017, 2018a).

## Highlights

• Quantitative real-time imaging of glutathione in live cells
• Subcellular quantification of glutathione using chemical- or protein-based targeting
• Quantitative flow cytometry of glutathione in live single-cell suspension

## Graphical Abstract

**Figure:**
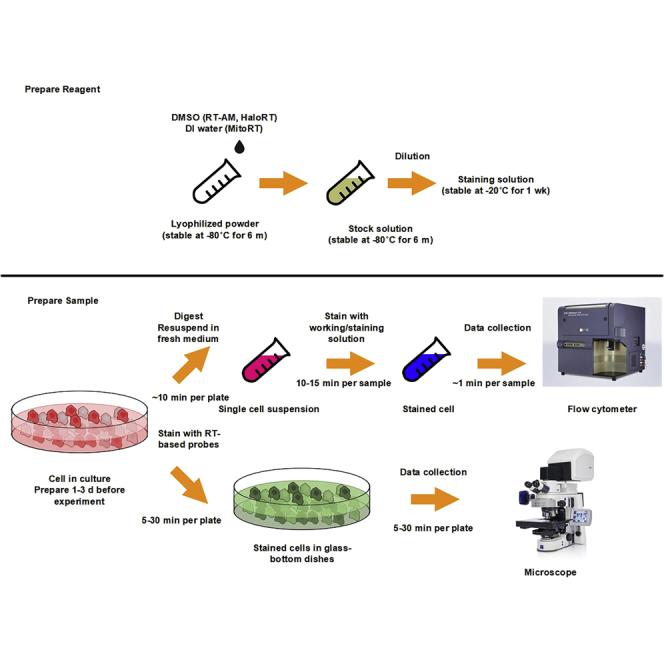

## Before You Begin

### Cell Culture

**Timing: 1–7 days before the experiment**

All cell lines are purchased through ATCC unless otherwise specified.

***Note:*** We used HeLa cells, HT-1080 cells, COS-7 cells, HepG2 cells, 3T3-L1 cells, PANC-1 cells, MOML-14 cells, primary hepatocytes from BALB/c mice, and hESC-derived neurons for our tests.

For imaging, cells should be cultured on a ***glass-bottom dish*** or equivalent; for flow cytometry, cells should be prepared as ***single-cell suspension***.

1. Adherent cells (e.g., HeLa, HT-1080, 3T3-L1, COS-7, PANC-1, PANC-28 cells):
  a. Cells are cultured in T-75 flask in 10% FBS supplied DMEM with 1% PS. (penicillin streptomycin antibiotic, see below list of reagents) at a 37˚C incubator with 5% CO_2_.
  b. The typical seeding density is 2.0×10^6^ cells, and cells grow to confluence in 2–4 days.
  c. Cells are passed regularly in the same flask.
  d. Cells used for tests are typically within 2 months of culture and <15 passages.
  e. Decreasing the intracellular GSH levels with BSO treatment typically requires cysteine free culture medium throughout the experiment.
  f. Other conditions that involves low intracellular GSH levels may also utilize cysteine free medium.

***Note:*** HaloRT-based subcellular GSH quantification requires transfection with appropriate organelle-specific HaloTag plasmids. Refer to Key Resources Table for details.

2. Non-adherent cells (e.g., MOLM-14 cells):

Cells are cultured in T-75 flask in 10% FBS supplied RPMI 1640 with 1% PS at a 37˚C incubator with 5% CO_2_. The typical seeding density is 2.0×10^6^ cells, and cells grow to confluence in 4–7 days. Cells are passed regularly in the same flask. Cells used for tests are typically within 2 months of culture and <15 passages.

3. Primary cells (e.g., primary hepatocytes):

Cells are collected from mouse primary tissue and kept in DMEM before use. Different primary cells may be adopted using different protocols. Please refer to the corresponding references for details. For primary hepatocyte extraction protocol, please refer to reference (Jiang et al., 2017).

4. Other cells (e.g., hESC-derived neurons):

Cells are kept in suitable medium such as neurobasal medium for neurons before use. Please refer to corresponding references for details.

## Key Resources Table

**Table:** 

REAGENT or RESOURCE	SOURCE	IDENTIFIER
**Bacterial and Virus Strains**		

Halo-Cyto Plasmid	Addgene	124314
Halo-MTS Plasmid	Addgene	124315
Halo-KDEL Plasmid	Addgene	124316
Halo-NLS Plasmid	This article	Please request from the authors

**Chemicals, Peptides, and Recombinant Proteins**		

RT-AM	Kerafast.com	EBY001
RT	Kerafast.com	EBY002
MitoRT	Kerafast.com	EBY003
HaloRT ligand	Kerafast.com	EBY004
DMEM	Thermo Fisher	10569044
Cysteine free DMEM	Thermo Fisher	21013024
Earle's Balanced Salt Solution (EBSS)	Invitrogen	14155063
Hanks' Balanced Salt Solution (HBSS)	Invitrogen	24020117
RPMI 1640	Thermo Fisher	72400047
Neurobasal medium	Thermo Fisher	10888022
FBS	Thermo Fisher	10099141
PBS	Thermo Fisher	10010023
Hepatocyte wash medium	Invitrogen	17704024
Percoll	Sigma	P4937
Wiliams E medium	Invitrogen	12551
Penicillin streptomycin 10,000 U/mL	Thermo Fisher	15140122
Anhydrous DMSO	Sigma	D8418, CAS: 67-68-5
Poly-D-lysine solution	Sigma	A-003-M
DL-Buthionine sulfoximine (BSO)	Sigma	19176, CAS: 5072-26-4
N-Methylmaleimide (NMM)	Sigma	389412, CAS: 930-88-1
Diethyl maleate (DEM)	Sigma	D97703, CAS: 141-05-9
H_2_O_2_	Sigma	216763, CAS: 7722-84-1
Trypan blue solution 0.4%	Sigma	93595, CAS: 72-57-1
Trypsin-EDTA 0.25%	Thermo Fischer	25200056
Glutathione ethyl ester	Sigma	G1404
Sodium hydroxide (NaOH) solution 1.0 N **Caution:** hazardous chemical, irritable, corrosive	Sigma	S2770, CAS: 1310-73-2

**Critical Commercial Assays**		

Packed cell volume (PCV) cell counting tubes	Sigma	Z760986
Pierce™ BCA Protein Assay Kit	Thermo Fischer	23227

**Experimental Models: Cell Lines**		

HeLa	ATCC	CCL-2
HT-1080	ATCC	CCL-121
COS-7	ATCC	CRL-1651
HepG2	ATCC	HB-8065
3T3-L1	ATCC	CL-173
PANC-1	ATCC	CRL-1469
MOML-14	Gift from Dr. Conneely at Baylor College of Medicine	N/A
primary hepatocytes from BALB/c mice	Gift from Dr. Moore at Baylor College of Medicine	N/A
hESC-derived neurons	Gift from Dr. Maletić-Savatić at Baylor College of Medicine	N/A

**Software and Algorithms**		

Fiji ImageJ	https://fiji.sc/	N/A
FlowJo	FlowJo LLC, a BD company	V10
Python	https://www.python.org/download/releases/2.7/	V2.7
Automated ratiometric analysis	https://github.com/jinwanglab/	N/A

**Other**		

35-mm glass-bottom dish	Denville scientific	T1121
Nunc Lab-Tek 8-well chamber slide	Thermo Fisher	154453
BD Polystyrene round bottom tubes 5 mL	BD Biosciences	352052
Zeiss LSM 780/880 Airyscan confocal Microscope system with live imaging chamber	Zeiss	LSM780, LSM880
Olympus FV1000 confocal microscope	Olympus	FV1000
BD LSRII Fluorescence-activated cell sorter	BD Biosciences	LSR II

## Materials and Equipment

### Stock Solutions

• To prepare the stock solution, dissolve the lyophilized RT-AM/RT probe powder in 10 μL of anhydrous DMSO to make a 5 mM stock solution.
• For HaloRT ligand, dissolve the lyophilized HaloRT ligand powder in 10 μL anhydrous DMSO to make a 5 mM stock solution.
• For MitoRT probe, dissolve the lyophilized MitoRT probe powder in 10 μL of deionized water (DI water) to make a 5 mM stock solution.

**CRITICAL:** DMSO is hygroscopic. DMSO with significant amount of water can hydrolyze RT-AM. It is highly recommended to dissolve RT-AM in anhydrous DMSO.

**CRITICAL:** DMSO can react with MitoRT and cannot be used as the solvent for MitoRT.

• Stock solution is stable at −80°C for up to 6 months. Make small aliquots if necessary.

### Staining Solutions

• To prepare the staining solution, typically, the stock solution is diluted with its corresponding solvent (DMSO for RT-AM and HaloRT ligands, DI water for MitoRT) 50 times to 100 μM.
• Staining solution can be stored at −20°C for 1 week. Make small aliquots if necessary.

### Working Solutions

• To prepare working solution, typically, the staining solution is further diluted with desired imaging buffer (fresh cell culture medium or other appropriate buffers) 100 times to a final probe concentration of 1 μM.

***Note:*** For RT-AM and HaloRT ligand, make sure to add the imaging buffer (aqueous phase) into the staining solution (organic phase), otherwise, precipitation of the fluorescent probes may happen. If precipitation occurs, centrifuge at 3,000 × *g* for 3 min and keep the supernatant. This may lead to inconsistent probe concentration but should not interfere with most measurements.

***Note:*** PBS is generally not advised for live cell experiments as the GSH level usually changes when cells are exposed to PBS for more than 5 min.

### Other Solutions Used in This Protocol

• GSH standard solution can be prepared by dissolving GSH powder in PBS or DI water. Typical concentration of GSH standard solution is between 0.5–50 mM in final solution. Adjust pH to 7.4 by adding NaOH solution when directly dissolve GSH powder in DI water. GSH solution should be made fresh each time to minimize errors from undesired oxidation.
• H_2_O_2_ working solution can be prepared by diluting from the original 30% solution using PBS to the desired concentration. The working solution needs to be made fresh each time.

**CRITICAL:** GSH and H_2_O_2_ are both unstable due to its redox active chemical properties. Make fresh solution each time and limit their exposure to air will reduce potential errors during quantification. GSH and H_2_O_2_ solutions are primarily used for calibration thus will determine the overall performance of this protocol.

• BSO stock solution (100 mM) can be prepared by dissolving BSO powder in PBS or DI water (preferred). BSO working solution can be prepared by diluting with the appropriate solvent or buffer.
• NMM stock solution (100 mM) can be prepared by dissolving NMM powder in anhydrous DMSO. NMM working solution can be prepared by diluting with DMSO.
• DEM stock solution (100 mM) can be prepared by diluting liquid DEM in anhydrous DMSO. DEM working solution can be prepared by diluting with DMSO.
• BSO, NMM and DEM stock solution can be stored at −20°C for 3 months. Make small aliquots if necessary.

### Microscope Setup

• Turn on confocal microscope, pre-warm laser light source and live imaging chamber.
• Choose the desired objective (typically 40× or 63× 1.4 NA oil objective is preferred), filter sets or detector wavelengths (for Zeiss LSM 780/880: green channel: 488 nm excitation, 499–615 nm emission; blue channel: 405 nm excitation, 418–495 nm emission; for filter-based Olympus confocal microscopes: green channel: 488 nm excitation, 575–620 nm emission filter; blue channel: 405 nm excitation, 430–470 nm emission filter). Apply one drop of the appropriate imaging oil onto the objective if using an oil immersion objective.
• Adjust gain and offset of each channel to satisfy the whole dynamic range of the experiment by imaging a positive (e.g., transient 200 μM GSH-ester treated cells) and negative control (e.g., 48–72 h 500 μM BSO treated cells) first.

***Note:*** The setting for Zeiss confocal microscopes provides the optimal signal to noise ratios on our hands.

### Slides and Imaging Dish Preparation

• Coat glass slides with poly-D-lysine and dry under 18°C–25°C. The coated slides can then be used for beads (for calibration) and non-adherent cell imaging. Alternatively, coated glass slides can be used to culture adherent cells and used for imaging similar to glass-bottom dishes.
• For glass-bottom dish or chamber slides, no additional steps are needed before imaging.
• Clean the bottom of imaging container with appropriate tools right before imaging to ensure the imaging quality.

### Flow Cytometer Setup

• Turn on BD LSRII flow cytometer, choose the desired filter sets: blue channel: Pacific Blue (405 nm excitation, 450/50 nm emission); green channel: PE (488 nm excitation, 575/26 nm emission).

***Note:*** DAPI channel usually uses 380 nm excitation and 450/50 nm emission, which is not optimal but still feasible. FITC channel is generally not advised because the emission filters do not match well.

• Adjust gain of each channel (including forward scatters, or FSC, side scatters, or SSC, green and blue channel) to satisfy the whole dynamic range of the experiment by running positive and negative control samples first.

***Alternatives:*** Other commercially available confocal microscopes and flow cytometers with the appropriate optical filters can also be used for this protocol.

## Step-By-Step Method Details

### Cell Sample Preparation

**Timing: 2–3 days**

In this step, sample staining and pretreatment for different scenarios are described in details. For best quantitative results, prepare control samples as described. For HaloRT-based probes, transfect cells with the appropriate plasmid is required.

#### Cell Transfection (for HaloRT-Based Experiment Only)

**Timing: 2–3 days**

1. Seed the cells 24 h before transfection at desired density in an appropriate container (2.0×10^6^ cells per sample in a glass-bottom dish for imaging; 1.0×10^5^ cells per well in a 12-well plate for flow cytometry).
2. Transfect cells with desired HaloTag plasmids using Lipofectamine 3000 transfection reagent or other suitable reagents (e.g., for nuclear GSH measurement, use Halo-NLS plasmid for transfection, 500 ng DNA per glass-bottom dish/per well in a 12-well plate). Culture the cells for 24–48 h.

***Alternatives:*** a stable cell line expressing desired HaloTag protein can be generated.

#### Drug Treatment

**Timing: 1 min to 3 days**

3. For GSH synthase inhibitor BSO treatment, add the appropriate amount of BSO to the cell culture medium while seeding the cells. Culture for 48–72 h. Cysteine free culture medium should be adopted for cell culture and for the following measurements.

***Note:*** BSO will partially affect cell growth when the seeding density is low. To best maintain the viability of cells, use a higher seeding density.

***Note:*** BSO treatment will take 72 h to reach the maximum effect. BSO Treatment for 24 h and 48 h will result in approximately 70% and 90% of the maximum effect, respectively.

***Note:*** The efficacy of BSO to reduce intracellular GSH levels is highly cell type dependent. Typically drug resistant cancer cells require much higher doses of BSO compared with other cells. To select the best BSO concentration range, pilot experiments are recommended. A starting concentration range of 50–100 μM BSO can be tested in most cultured cells.

4. For short term drug treatment, add the desired compounds to the culture medium and incubate as instructed by the distributor. Typically, 10–100 μM NMM/DEM can reduce GSH concentration by 50% or more in 30–60 min.
5. For monitoring GSH changes during transient compound treatment such as H_2_O_2_ or NMM, add the drugs to the sample while acquiring signals or just prior to signal acquisition.

***Note:*** To minimize changes to the culture environment, it is advised to add the drug at a relatively high concentration, e.g., 100× or 10×. It is also advised to avoid directly adding DMSO containing stock solution into water phase during signal acquisition as the release of heat from solvent mixing may instantly kill the cells and create artifacts.

***Note:*** Some cells are highly capable of defending against external stimulants. In this case, the changes of the GSH concentrations may be subtle, or the changes may be reversed within a short period of time. Thus, real-time imaging can be advantageous over other signal acquisition methods for recording such transient events.

#### Cell Staining

**Timing: 8–60 min per sample**

6. Replace cell culture medium with working solution containing RT-AM/MitoRT/HaloRT ligand. For adherent cells, this step could be done through aspiration of culture medium and then addition of the working solution; for non-adherent cells, a centrifugation step is required before removing the culture medium, and a resuspension step after addition of working solution.
7. For flow cytometry experiments, all cells need to be in single-cell suspension prior to staining. For adherent cells, use trypsin to digest cells and resuspend into fresh culture medium. The staining process follows the non-adherent cell staining protocol with centrifugation and resuspension steps.
8. Incubate the cells in the working solution for **8–10 min** at **18°C–25°C** for RT-AM stained cells; **15–30 min** at **37°C** for MitoRT and HaloRT ligand stained cells. Alternatively, longer incubation (30–60 min) at room temperature for MitoRT and HaloRT ligand stained cells may be used in cases where heating devices are unavailable.

**CRITICAL:** Temperature of staining will influence the distribution homogeneity of RT-based probes. Generally, a lower temperature will induce less aggregation or clearance of probe thus lead to more homogeneous probe loading. For organelle targeting probes, the targeting mechanism will lock desired probes in place with the protein, a higher loading temperature will facilitate clearing of non-specific probes in undesired organelles.

9. For non-adherent cells (including polystyrene bead-based calibration), place 10 μL of suspension containing stained cells onto a coated glass slides, and then cover with cover glass. The sample is then ready for imaging.

***Note:*** The sample may dry out in 15 min, please mind the timing. Alternatively, seal the sample with a specific O-ring to prevent drying.

**CRITICAL:** Washing steps should be generally avoided for RT-AM and MitoRT stained cells when confocal imaging is used for signal acquisition because confocal imaging can easily distinguish between intracellular signal and excess fluorophore in solution and a washing step may induce additional stress to cells.

***Note:*** Washing step will influence cell state and lead to rapid changes in cytosolic redox conditions. We have observed 20% GSH concentration fluctuation due to washing. This is especially important for imaging experiment because 1) sampling in typically limited in confocal imaging experiment; 2) imaging experiment is typically designed for capturing fast yet subtle changes compared to other type of experiments thus imaging is more prone to such bias.

10. Wash cells with fresh culture medium in HaloRT ligand stained cells, and incubate cells at 37°C for 15–30 min. The washing step can be repeated one more time if signal to noise is still not optimal after one washing step. For confocal imaging experiments, the washing step may be skipped if the signal to noise is above 5.

***Note:*** HaloRT ligand require long incubation for cells to clear non-specific binding of probes. The signal is also retained better than chemical targeting and thus allow possible longer recovery time to overcome the fluctuation caused by washing.

11. Wash cells with fresh culture medium can be skipped for flow cytometry experiments. If the signal to noise is below 5, washing steps may help.

### Signal Acquisition

**Timing: 2–15 min per sample**

In this step, quantitative fluorescent signal acquisition of individual samples using confocal microscope or flow cytometer are described. Signal acquisition steps should immediately follow staining steps for each sample. Generally, avoid staining and measuring in batches as GSH and the probes are highly dynamic, and that staining relies on fast reversible chemical reactions.

#### Confocal Imaging

**Timing: 5–15 min per sample**

12. Use sequential imaging on two channels. Use line-switching mode when possible, especially for imaging within smaller organelles such as mitochondria. Z-stack is generally not advised due to the slow speed. Alternatively, if sequential imaging is not available or too slow, a more complicated calculation should be performed when processing the data. Keep the imaging conditions exactly the same as the conditions used for the calibration curve when absolute quantification is needed.

***Note:*** Generally, ratio between signals from blue and green channels is linear to the GSH concentration. However, if the imaging condition is not ideal, the linearity will not hold and thus a complex ratio (R-R_min_)/(R_max_-R) should be used, where R is the measured ratio, R_max_ is the ratio with maximum signal (need 50 mM or higher of GSH for measurement), R_min_ is the ratio with minimum signal (need 0.5 mM or lower combined GSH, cysteine, and other thiols for measurement).

13. For RT-AM based global GSH quantification in live cells, we advise a short imaging time due to the relatively fast probe clearance especially in cancer cell lines. In cases when long monitoring is needed, ensure applying an appropriate non-treated, stained cell sample as a control. Alternatively, the HaloRT strategy can be applied for long-term monitoring experiments.
14. For MitoRT based mitochondria GSH (mGSH) quantification in live cells, a higher magnification may be needed. Some super-resolution imaging method (such as stimulated emission depletion super-resolution imaging or airy- scan) may be used, but the quantification may be compromised.
15. For HaloRT-based organelle-specific GSH quantification in live cells, the imaging time can be extended.

**CRITICAL:** Stain the next sample while waiting for imaging to complete. Keep staining time the same for each sample.

16. For quantification or semi-quantification purpose, please refer to the calibration section for details about creating standard curve for calibration; for qualitative assessment of samples, standard curve can be skipped.

***Note:*** Please keep laser intensity low to avoid light toxicity and fluorophore bleaching.

***Note:*** Please have an appropriate probe only control for live monitoring experiments to test the probe clearance speed in your cells of interest.

***Note:*** Trypan blue can be used to quench cell surface non-specific stain in cases where cell surface fluorescence is high and the signal to noise is below 5. For quality purpose, signal to noise ratio of 2 is considered low and signal to noise above 6 is considered high. Please refer to Troubleshooting for additional information regarding abnormal imaging results.

#### Flow Cytometry

**Timing: 2–5 min per sample**

17. Typically, cells are dispersed into single-cell suspension (500 μL in a 5 mL polystyrene tube) and brought to the flow cytometer prior to staining.

**CRITICAL:** The cells are then stained on-site by adding the probe (5 μL staining solution), vortex, and incubate for required time (for RT-AM staining, 8–10 min; for MitoRT and HaloRT staining,15–30 min) and immediately analyzed. Keep staining time the same across all samples to minimize variations.

18. The gain settings should allow signal to noise >3 in both channels for both positive and negative control samples compared with the non-stained control sample.
19. It is advised to acquire signals from at least 10,000 cells per sample with an optimal of 30,000 or more cells.
20. For quantification or semi-quantification purpose, please refer to the calibration section for details about creating standard curve for calibration; for qualitative assessment of samples, standard curve can be skipped.

***Note:*** MitoRT may partially lose organelle targeting ability during single-cell suspension preparation from adherent cells, because part of the mitochondria loses activity and polarity during cell detachment process thus interrupts the charge based chemical targeting mechanism. To confirm, an imaging flow cytometer can be used. Please refer to Troubleshooting for additional information.

### Data Processing: Imaging Data Processing

**Timing: 0.5–2 h**

21. In Fiji ImageJ, open the acquired images, duplicate each fluorescence channel into single frames.
22. Run background subtraction using the rolling ball algorithm with diameter 100 (depending on the actual images, different background subtraction algorithm can be used).
23. Run image calculation and divide the blue channel image by the green channel image to generate a ratio map. Change LUT (lookup table) for favorable visualization, e.g., physics. Save all images as desired.
24. Mask the ratio map with regions-of-interest when necessary before quantitative analysis, especially in organelle-specific GSH measurements.
25. Record absolute ratio data from individual cells/organelles in all images from the dataset. Manual picking of region of interest may be necessary.
26. Compare with standard curve to deduct the absolute GSH concentrations when performing quantification or semi-quantification (please refer to the calibration section for standard curves).

***Note:*** Please refer to literature for illustration and examples.(Jiang et al., 2017, 2018a)

***Note:*** Image processing in Fiji ImageJ (steps 21–23) can be partially automated by scripts. However, ratio data collection and quantitative analysis still require manual work. Example script can be found on GitHub: https://github.com/jinwanglab/RT-Imaging-Analysis

### Data Processing: Flow Cytometry Data Processing

**Timing: 0.5–1 h**

27. In FlowJo, open the data files. Gate by FSC and SSC to define the single-cell population.
28. Export the blue and green channel signal in the single-cell population to a .csv file.
29. Calculate the ratio in each cell by divide signal from the blue channel by that from the green channel. Remove outliers that are 4 standard deviations away from the mean.
30. Compare with standard curve to deduct the absolute GSH concentrations when performing quantification or semi-quantification.

***Note:*** Statistical data analysis from step 29 can be automated by scripts. Example script can be found on GitHub: https://github.com/jinwanglab/RT-FACS-Analysis

### Calibration against Lysate-Based Assay (Preferred Method)

**Timing: 2–3 days**

31. Prepare two identical groups of cells by treating with increasing concentrations of BSO (e.g., 0–500 μM for HeLa cells) for 48–72 h. One group of cells should be cultured in a 12-well or 6-well plate for lysate-based assay, the other group of cells should be cultured in the appropriate containers as described above.
32. Take the group of cells cultured for lysate-based assay, measure GSH using standard Ellman’s assay(Rahman et al., 2006) or liquid chromatography-mass spectrometry-based assay(Jiang et al., 2017; Squellerio et al., 2012).
33. Measure the other group of cells using any of the probe with the corresponding protocol described above. Analyze and record ratio value for each sample.
34. Compare assay results with ratio and fit with an appropriate function.

***Note:*** The ratio and GSH concentration may not follow linear relationship depending on the actual experimental conditions. Please refer to the literature(Jiang et al., 2015, 2017) for detailed explanation and function deduction in cases where the fitting significantly deviates from linear.

***Note:*** Even for semi-quantitative/qualitative experiments, it is still recommended to perform this calibration experiment to assess GSH levels based on the ratio readouts.

### Calibration Using Standard Solutions (Only Applicable for Imaging)

**Timing: 0.5–1 h**

35. Add 15 μm polystyrene beads suspension into RT solution. Typically, add 1 μL of beads per 100 μL of RT solution.

**CRITICAL:** Prepare fresh GSH stock solution at 200 mM in PBS. Add 1.0 N NaOH solution to adjust pH to 7.4.

36. Serial dilute GSH stock solution to desired concentration, for example, 20, 10, 5, 2.5, 1.25, 0.625 mM of GSH.
37. Mix equal amount (typically 4 μL) of GSH with RT solution, incubate at 18°C–25°C for 10 min.
38. Add the above solution onto a coverslip, cover with slide glass, and take images. Make sure to pipette the mixture to prevent beads from aggregating into one spot.
39. To measure R_min_, use 0.01 mM of final concentration of GSH; to measure R_max_, use 100 mM final concentration of GSH.
40. Calculate the ratio from each group, plot (R−R_min_)/(R_max_−R) against GSH concentration, a linear relationship can be established as a standard curve for comparing results.

***Note:*** All conditions for calibration should be kept the same with the actual imaging experiments, including objectives, temperature, magnification, Gain/PMT, offset, filter, laser intensity settings.

***Note:*** This calibration method pairs with the RT-AM based imaging experiments.

***Note:*** The GSH standard solutions can be oxidized in air. It is advised to perform this experiment as fast as possible or use nitrogen for protection. Always start with the lowest concentration of GSH because it is more prone to oxidation errors.

***Note:*** This calibration method can also be used for HaloRT-based imaging experiments, in which the RT solution should be replaced with the HaloRT solution (made from recombinant HaloTag protein reaction with the HaloRT ligand).

***Note:*** This calibration method is **not** suitable for MitoRT based imaging experiments, possibly because the microenvironment for MitoRT is mitochondria cannot be faithfully simulated using an aqueous solution.

### Cell Volume Estimation

**Timing: 5–60 min**

The cell volume data can be used to calculate the absolute intracellular GSH concentrations from bulk lysate- based measurements. The data is needed for lysate-based assay calibration purpose.

#### PCV Tubes

**Timing: 5–10 min**

41. Prepare single-cell suspension as described above.
42. Transfer 1 mL of cell suspension into a PCV tube.
43. Spin multiple samples for 1 min at 2,500 × *g* in a standard microcentrifuge.
44. Record pellet volume.
45. Count the cell number using a hemocytometer.
46. Calculate individual cell volume from the cell number and the total volume.

#### Imaging Measurement

**Timing: 5–10 min**

47. Take a z-stack image dataset in transmitted light channel.
48. Measure the length, width, and height of the cell in the z-stack image dataset.
49. Calculate the average cell volume from multiple datasets.

#### Protein Concentration-Based Estimation

**Timing: 60 min**

50. Use standard bicinchoninic (BCA) assay or other assays to measure the protein concentrations of cells. The cells should be prepared the same way as the calibration group. Other protein quantification methods such as Bradford assays can also be used as long as the dynamic range fits.
51. Estimate cell volume using available data on the corresponding protein concentration per unit volume.

## Expected Outcomes

### Expected Absolute GSH Concentration

The typical concentration range of intracellular GSH is 1–10 mM, where RT-based probes show optimal performance. In cancer cells or highly metabolic active cells, the GSH concentration is usually close to the high-end; while in certain drug treated cells and certain organelles such as in the ER, the GSH concentration is closer to the low-end. For cultured cells, low passage cells showed slightly lower overall GSH concentration. Cells may exhibit day-to-day or batch-to-batch differences in their concentration, sometimes the differences can be as high as 50%. Some example results that we measured previously using this protocol are summarized in the table:

**Table:** 

Cell Type	Subcellular Location	Drug Treatment	Expected Concentration	Probe
3T3-L1	global	N/A	4.6±0.9 mM	RT-AM
HepG2	global	N/A	6.2±1.9 mM	RT-AM
HeLa	global	N/A	4.6±0.8 mM	RT-AM
PANC-1	global	N/A	6.4±1.8 mM	RT-AM
PANC-28	global	N/A	6.6±1.7 mM	RT-AM
hESC-derived neuron	global	N/A	8.0±0.5 mM	RT-AM
HT-1080	global	N/A	11.8±1.8 mM	RT-AM
HeLa	ER	N/A	~1.0±0.5 mM	HaloRT
HeLa	nucleus	N/A	6.3±1.2 mM	HaloRT
HeLa	cytosol	N/A	6.4±1.3 mM	HaloRT
HeLa	mitochondria	N/A	4.9±0.8 mM	HaloRT and MitoRT
HeLa	global	500 μM BSO treated (72 h)	~0.8±0.2 mM	RT-AM

#### Stable Readout Time Window Before Probe Clearance

The probes rely on fast reversible chemical sensing reaction for GSH quantification. Due to the nature of reaction and cell intrinsic clearance pathways, long-term incubation leads to probe clearance and unstable readouts. We recommend keeping the readout time short and consistent whenever possible. The stability of signals in different conditions that we have tested are summarized in the table:

**Table:** 

Probe	Cell type	Temperature	Stable time window
RT-AM	HeLa	18°C–25°C	~30–60 min
RT-AM	HeLa	37°C	~5–10 min
MitoRT	HeLa	37°C	~15–20 min
HaloRT	HeLa	37°C	~30–45 min
RT-AM	Primary hepatocytes	18°C–37°C	~15–20 min
MitoRT and HaloRT	Primary hepatocytes	18°C–37°C	~30–60 min
RT-AM	hESC-derived neurons	18°C–37°C	~30–120 min

***Note:*** There have been reports of longer monitoring using RT-AM in certain cells (HaCaT cells) for up to 400 min with stable signals.(Rothmiller et al., 2018)

#### Live Monitoring Experiments in Confocal Imaging

Cells may drift during live monitoring thus causing artifacts. Re-focus when necessary during time-lapsed imaging.

Due to potential probe clearance, live monitoring results may show increasing blue/green ratio over time, especially in cancer cells and metabolically active cells. Proper control should be designed for long-term tracking experiments.

Addition of drugs during live monitoring may cause solvent mixing heat exchange, reagent slow diffusion, and cell morphology change artifacts. Use cautions when adding drugs, avoid directly adding drugs on top of cells in the field of view.

***Note:*** Avoid using live imaging buffer that contains antioxidants (e.g., ProLong Live Antifade Reagent, Thermo Fisher P36975; Live Cell Imaging Solution, Thermo Fisher A14291DJ) for imaging experiments as it may change the redox environment of cells.

#### Single Point GSH Quantification

The cell seeding density has significant influence on the intracellular GSH concentration. Generally, dense areas tend to have high GSH concentrations.

Washing steps, especially washing with PBS multiple times, can cause significant changes of intracellular GSH concentrations.

Different batches/passages of cells may have significantly different GSH concentrations. The difference can be as large as 2- to 3-fold.

## Limitations

We explained some limitations of the RT probes in a recent correspondence.(Jiang et al., 2018b) “Despite the advantages of RT, it is important to understand its limitations to obtain meaningful measurements. The sensing moiety of RT essentially reacts with all the small molecule thiols, including GSH, Cys, and even reduced trypanothione, but to a lesser extent toward protein thiols, probably due to steric hindrance or charge interactions. RT can be claimed as a GSH probe only when GSH is the dominant form of small molecule thiols and in the mM range. In the case that the total concentration of small molecule thiols is in the μM range, RT cannot perform accurate measurements since it is outside the dynamic range. If Cys were in the mM range in cells, RT would respond to Cys as well, although we are unaware of a situation like this in mammalian cells. Therefore, we advise users to either measure relative concentrations of Cys and GSH in the system in which they use by HPLC methods or search for available literature data, such as BioNumbers.org, before using the RT probe.”

Long-term tracking of GSH using RT-based probes may not be ideal in certain cell lines due to the clearance of probes through ABC transporters, especially in some cancer cell lines. We recommend multiple short time-lapsed imaging for cells with fast probe clearance.

Certain cells may experience significant outer membrane accumulation of free probe causing strong background. Higher spatial resolution or certain washing and masking steps may be helpful.

Laser-based technology with high spatial resolution is generally required to achieve high signal to noise and good quantitative results. Generally, epi-fluorescence microscopy may not distinguish signals from the background, unless specific optical settings or algorithm are adopted.

Ratiometric quantification relies on the corresponding standard curve. In some cases, the standard curve is unavailable or difficult to obtain. Therefore, semi-quantitative or qualitative measurement can be used.

## Troubleshooting

### Problem 1

Signals primarily accumulate on cell outer membrane.

### Potential Solution

**Figure 1:**
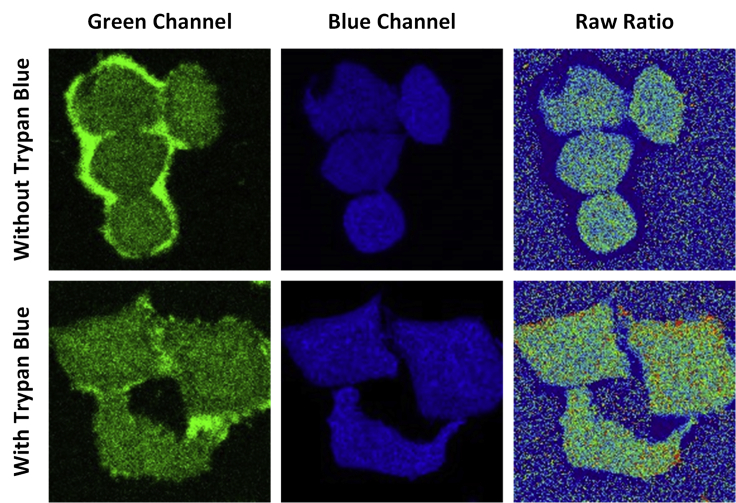
Illustration of Trypan Blue Quenching HeLa Cell Outer Membrane Fluorescence The two channels have different propensity of lingering signal to the outer membrane, thus will lead to artifacts. Trypan blue partially resolves this issue but will also change the raw ratio, resulting in the need for a new calibration curve for quantification.

Please check if the cells have been passaged for too many times. Generally, this could be avoided using low passage cells and washing thoroughly each time during passage. For certain cells with extremely sticky membrane, additional washing steps may be helpful. Alternatively, adding non-permeable fluorescent quenching reagent may also help, such as trypan blue (Fig. 1). However, it will lower the overall signal to noise and alter the quantification curve.

### Problem 2

No signals can be detected.

### Potential Solution

First, check the fluorescent channel selection to make sure the blue (Pacific Blue, 405 excitation) and green (PE, 488 excitation) channels are selected correctly; second, check the gain setting for each channel (including FSC and SSC) and adjust slowly across the whole dynamic range. Contact flow cytometry service personnel when necessary.

### Problem 3

No signal differences between samples.

### Potential Solution

Use positive control (200 μM GSH-ester treated cells) and negative control (500 μM BSO treated cells) to acquire data and compare ratios with non-treated cells. HeLa cells are advised to be used as a control cell line for pilot study, as we have extensively demonstrated different RT-based probes can be successfully applied for monitoring GSH changes under various conditions, and that HeLa cells are readily available in most biology labs. Prepare fresh staining solution for measurements. Check if cysteine free medium should be used for the low GSH group.

### Problem 4

Inconsistent signals between repeats.

### Potential Solution

Certain cell lines (especially cancer cell lines) suffer from fast probe clearance, resulting in loss of signal in a relatively short period. To test signal acquisition time window, try a different staining time between 5 and 30 min, then measure the same sample 0, 10, 20, and 30 min post staining with acquisition time <2 min for each measurement. If none of the conditions can provide stable signals, try lowering the temperature to prevent probe clearance, or by adding ABC transporter inhibitors such as GF120918 or probenecid.

### Problem 5

Standard curve quantification does not correlate with biochemical/HPLC assay.

### Potential Solution

BSO may deplete GSH differently across different cell types, therefore, different dilution factors should be tested when testing on a new cell type. Cell volume estimation may introduce the biggest error in the calculation due to the relatively high variance with the current method.

Alternatively, a semi-quantitative standard curve may be used in which the RT-based measurement can be correlated with biochemical/HPLC direct readings.

## Resource Availability

### Lead Contact

Further information and requests for resources and reagents should be directed to and will be fulfilled by the Lead Contact: Jin Wang, wangj@bcm.edu

### Materials Availability

All materials are commercially available unless otherwise specified.

### Data and Code Availability

All original data provided by this study is available through the lead contact.

All code provided by this study is available through GitHub: https://github.com/jinwanglab/
